# *Mycobacterium* Phage Butters-Encoded Proteins Contribute to Host Defense against Viral Attack

**DOI:** 10.1128/mSystems.00534-20

**Published:** 2020-10-06

**Authors:** Catherine M. Mageeney, Hamidu T. Mohammed, Marta Dies, Samira Anbari, Netta Cudkevich, Yanyan Chen, Javier Buceta, Vassie C. Ware

**Affiliations:** a Department of Biological Sciences, Lehigh University, Bethlehem, Pennsylvania, USA; b Department of Chemical and Biomolecular Engineering, Lehigh University, Bethlehem, Pennsylvania, USA; c Department of Bioengineering, Lehigh University, Bethlehem, Pennsylvania, USA; University of Hawaii at Manoa

**Keywords:** *Mycobacterium*, defense mechanisms, mycobacteriophage, prophage, viral defense

## Abstract

A diverse set of prophage-mediated mechanisms protecting bacterial hosts from infection has been recently uncovered within cluster N mycobacteriophages isolated on the host, Mycobacterium smegmatis mc^2^155. In that context, we unveil a novel defense mechanism in cluster N prophage Butters. By using bioinformatics analyses, phage plating efficiency experiments, microscopy, and immunoprecipitation assays, we show that Butters genes located in the central region of the genome play a key role in the defense against heterotypic viral attack. Our study suggests that a two-component system, articulated by interactions between protein products of genes *30* and *31*, confers defense against heterotypic phage infection by PurpleHaze (cluster A/subcluster A3) or Alma (cluster A/subcluster A9) but is insufficient to confer defense against attack by the heterotypic phage Island3 (cluster I/subcluster I1). Therefore, based on heterotypic phage plating efficiencies on the Butters lysogen, additional prophage genes required for defense are implicated and further show specificity of prophage-encoded defense systems.

**IMPORTANCE** Many sequenced bacterial genomes, including those of pathogenic bacteria, contain prophages. Some prophages encode defense systems that protect their bacterial host against heterotypic viral attack. Understanding the mechanisms undergirding these defense systems is crucial to appreciate the scope of bacterial immunity against viral infections and will be critical for better implementation of phage therapy that would require evasion of these defenses. Furthermore, such knowledge of prophage-encoded defense mechanisms may be useful for developing novel genetic tools for engineering phage-resistant bacteria of industrial importance.

## Abstract (precis)

Many sequenced bacterial genomes, including those of pathogenic bacteria, contain prophages. Some prophages encode defense systems that protect their bacterial host against heterotypic viral attack. Understanding the mechanisms undergirding these defense systems is crucial to appreciate the scope of bacterial immunity against viral infections and will be critical for better implementation of phage therapy that would require evasion of these defenses. Furthermore, such knowledge of prophage-encoded defense mechanisms may be useful for developing novel genetic tools for engineering phage-resistant bacteria of industrial importance.

## INTRODUCTION

Mycobacteriophages—viruses infecting mycobacterial hosts—are of interest because they are useful in diagnostics of mycobacterial infections (1), the most notable of which is tuberculosis (TB), and additionally, can serve as genetic tools for mycobacteria (2–5). Most recently, engineered mycobacteriophages have been used in therapeutic applications to combat infections from antibiotic-resistant strains of Mycobacterium abscessus (6). To date, over 11,000 mycobacteriophages have been isolated, over 1,800 have been sequenced, and over 1,600 are available in GenBank (7, 8). Mycobacteriophages are a small subset of the estimated 10^31^ bacteriophages existing in the biosphere (9). Mycobacteriophages display high levels of genetic diversity and have been divided into 29 genomically similar clusters (A to AC) and a group of singletons with no close relatives (7, 10). Within several clusters, subclusters are defined as subgroups that share more extensive genomic similarities (7, 10). Although an increase in isolation and genomic characterization of mycobacteriophages has occurred recently, the void in knowledge about gene expression and function of mycobacteriophage gene products remains.

Most bacterial genomes contain prophages (11). The relationship between prophages and bacterial strains has shown numerous benefits to both the hosts and phages. Prophages confer many advantages to the host upon integration, such as enhanced fitness, reduction of mutation rates, selective advantages, and defense against additional viral attack (12). In this context, numerous mechanisms of defense have been recently discovered for *Pseudomonas*, *Mycobacterium*, *and Gordonia* prophages (13–16), with the expectation that prophage-mediated defense systems are likely widespread throughout the bacteria-phage world. These defense systems have biological impacts that include increasing fitness advantages for the host and influencing bacterial evolution (13). Intuitively, these defense systems have the potential to thwart phage therapy applications.

Cluster N phages have been investigated for prophage-encoded defense mechanisms that allow the host bacterium to resist attack by specific heterotypic phages (14). Different cluster N-specific defense systems were unveiled (14), with the prospect that additional defense systems in this phage group were yet to be discovered. Currently, 32 cluster N mycobacteriophage genomes are found in GenBank (8). Cluster N mycobacteriophages are characterized by small genomes (40.5 to 44.8 kbp) for mycobacteriophages (genome sizes range from 38.3 to 164.6 kbp) (7 [phagesdb.org], 14). Cluster N mycobacteriophages are capable of integration into the Mycobacterium smegmatis mc^2^155 *attB* site tRNA-Lys (MSMEG_5758) (14, 17).

Here, we focus on *Mycobacterium* phage Butters, which was isolated from soil on M. smegmatis mc^2^155. Butters is one of the smallest members of cluster N, with a genome of 41,491 bp (18), and contains 66 open reading frames (ORFs). The Butters genome can be divided into three regions (Fig. S1). Genes in the first region are rightward-transcribed, encoding structural genes such as capsid and tail proteins (genes *1* to *25*). The central portion of the genome (genes *26* to *40*) encodes two endolysins (lysin A and lysin B), a holin, genes used for integration and excision of the genome, and importantly, many genes with unknown functions. Within the central region of all cluster N genomes is the “variable region” (Fig. S1), which has considerable genomic variation among all cluster N phages (14). Finally, the third region includes rightward-transcribed genes (genes *41* to *66*) encoding proteins used in DNA maintenance and many of unknown function.

10.1128/mSystems.00534-20.1 FIG S1 Genome map of mycobacteriophage Butters with predicted functions depicted above the boxes. Genes are displayed above (rightward-transcribed) or below (leftward-transcribed). The genes are numbered according to the phamily number designated by Phamerator database Actino_Draft version 353 (6); the number of phamily members is indicated in the parentheses. Gene coloring is randomly produced by Phamerator. The gray shadow box corresponds to the central “variable region” (see Fig. 2A). Download FIG S1, TIF file, 0.3 MB. Copyright © 2020 Mageeney et al. 2020 Mageeney et al. This content is distributed under the terms of the Creative Commons Attribution 4.0 International license.

Cluster N mycobacteriophage prophage-mediated defense is a function of genes in the central variable region (14). Genes *30* and *31* are in the Butters variable region and were originally classified as orphams (i.e., genes with no known mycobacteriophage counterpart) prior to their discovery in a recently characterized cluster N phage, Rubeelu. However, their function remains unknown. These genes are among those expressed in a Butters lysogen (14), rendering them suitable candidates that mediate defense of the lysogen against heterotypic phages.

Two newly discovered defense systems in related groups of phages resemble the Butters gp30 and gp31 expression pattern and subcellular localization. Mycobacteriophage Sbash gp30 and gp31 (encoded by genes located in the central region of the Sbash genome) have no known homologues (15). These two proteins are expressed during lysogeny and encode a cytoplasmic protein (gp30) and a 4-pass transmembrane protein (gp31). The mechanism of action for these two proteins resembles the RexA/B system of coliphage Lambda; gp31 is located at the membrane, incoming phage attack by specific heterotypic phages (e.g., Crossroads) triggers gp30 activation, and the ion channel (gp31) is stimulated. Ion channel stimulation causes membrane depolarization and loss of intracellular ATP, which in turn, causes abortive infection of Crossroads. A similar RexA/B system has also been described for *Gordonia* phage CarolAnn (16). CarolAnn gp44 and gp43 are distantly related homologues of Sbash gp30 and gp31, respectively, but conserve predicted subcellular localizations (membrane [gp43] and cytoplasmic [gp44]). Heterotypic *Gordonia* phage Kita triggers a similar membrane depolarization mechanism. In each of these cases, the gene pair (*30*/*31* in Sbash and *43*/*44* in CarolAnn) was required to confer defense; expression of neither gene alone was sufficient for the defense phenotype. Although heterotypic phage proteins targeted in these two different systems are not conserved in sequence, it remains to be determined if any similarities exist in the mechanism of action for defenses encoded by Sbash and CarolAnn.

Here, we used bioinformatics analyses, heterotypic phage plating efficiency experiments, microscopy, and immunoprecipitation experiments to explore the roles of gp30 and gp31 in protecting a Butters lysogen from phage attack. Our results suggest that gp30 and gp31 interact and that gp31 may have an impact on the subcellular localization of gp30. Efficiency of plating data on M. smegmatis strains expressing gp30, gp31, or gp30 and gp31 combined show that PurpleHaze (subcluster A3) attack is completely abolished when gp30 is expressed alone, but infection is partially restored when gp30 is coexpressed with gp31. Moreover, for subcluster A9 phage Alma, viral attack is significantly inhibited by gp30, but no inhibition is observed when gp30 is coexpressed with gp31. Altogether, we propose that gp30-gp31 interaction is instrumental against specific viral attack. Further, since the proposed Butters gp30/gp31 system has no apparent effect on attack by subcluster I1 phage Island3 (but phage infection is significantly inhibited in a Butters lysogen), we suggest that a gp30-independent defense mechanism operates against this phage. Collectively, these data demonstrate that multiple defense mechanisms are encoded by the Butters prophage.

## RESULTS

### Bioinformatics analyses predict transmembrane domains for mycobacteriophage Butters gp31 but not for gp30.

Several bioinformatics programs were used to explore the prevalence, structural, and functional features of Butters gp30 (GenBank protein ID AGI12977.1) and gp31 (GenBank protein ID AGI12978.1). A BLAST search on the NCBI database (https://blast.ncbi.nlm.nih.gov/) resulted in hits to several *Actinobacteria* (including clinical isolates). *Actinobacteria* with orthologues of Butters gp30 and gp31 with greater than 40% amino acid identity are shown in Table 1. In all cases examined, the Butters gene *31* orthologue is the immediate downstream gene of the Butters gene *30* orthologue; synteny is therefore conserved. No putative functions were revealed for either protein by BLAST search.

**TABLE 1:** Orthologues of Butters gp30 and gp31 within *Actinobacteria*

*Actinobacteria* sp. strain GenBank accession no. (notes)	Coordinates (gene 30 orthologue)	Amino acid identity (gp30) (%)	GenBank protein accession no. (gp30)	Coordinates (gene 31 orthologue)	Amino acid identity (gp31) (%)	GenBank protein accession no. (gp31)
Mycobacterium abscessus *abscessus* 625 NZ_FSPH01000001.1 (clinical isolate, USA)	288451–289599	65.96	WP_050438738.1	287892–288458	81.91	WP_032667838.1
M. abscessus *abscessus* 599 NZ_FVTP01000002.1 (clinical isolate, USA)	581713–582861	65.96	WP_050438738.1	582854–583420	81.91	WP_032667838.1
M. abscessus (*abscessus*) 123 NZ_NQUM01000001.1 (clinical isolate, Shanghai, China)	281680–282828	65.96	WP_050438738.1	281121–281687	81.91	WP_032667838.1
M. abscessus *abscessus* Z58 NZ_JASW01000024.1 (clinical isolate, Hangzhou, China)	18408–19556	65.96	WP_050438738.1	19549–20115	81.91	WP_032667838.1
M. abscessus Z61 NZ_JASX01000040.1 (clinical isolate, Hangzhou, China)	13453–14601	65.96	WP_050438738.1	14594–15160	81.91	WP_032667838.1
M. abscessus G216 NZ_QXAG01000001.1 (clinical isolate, China)	16260191627167	65.96	WP_050438738.1	1627160–1627726	81.91	WP_032667838.1
M. abscessus (*massiliense*) A254 NZ_NQPL01000002.1 (clinical isolate, Shanghai, China)	80319–81467	65.96	WP_050438738.1	79760–80326	81.91	WP_032667838.1
*Mycobacterium* sp. DL99 NZ_SJOM01000007.1 (USA)	3292408–3293550	64.58	WP_135454704.1	3293543–3294104	69.15	WP_135454706.1
*Rhodoccoccus baikonurensis* NZ_BBBO01000007.1	1486–1947	47.17	WP_054780882.1 ^*a*^	2596–3165	65.96	WP_054780961.1
	1973–2584	45.96	WP_054780883.1 ^*a*^			

a A stop codon within the Butters gp30 orthologue yields two segments that map to distinct segments of Butters gp30.

**FIG 1:**
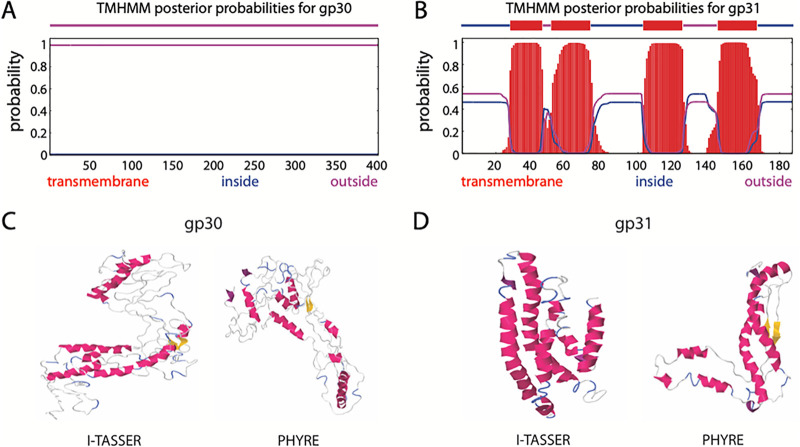
(A and B) Posterior probabilities for protein gp30 (A) and gp31 (B) as predicted by TMHMM (19, 20). The amino acid index is shown on the horizontal axis. The blue, purple, and red lines indicate the probability of an amino acid being located inside, outside, or within the cell membrane, respectively. Butters gp30 is predicted as a protein with domains outside the membrane (cytoplasmic). Butters gp31 is predicted to have 4-pass transmembrane domains (membrane protein). (C and D) Predicted secondary structures of proteins gp30 (C) and gp31 (D) using I-TASSER (21) and Phyre (22). The long, parallel, alpha helices of gp31 are characteristic of membrane proteins as predicted by TMHMM.

Next, Butters gp30 and gp31 were analyzed for transmembrane domains using TMHMM (19, 20). Butters gp30 was not predicted to have any transmembrane domains (TMDs) (Fig. 1A), while gp31 is predicted to have four (Fig. 1B). Two additional proteins, gp28 and gp21 (GenBank protein IDs AGI12975.1 and AGI12968.1, respectively), were analyzed by TMHMM and used as bioinformatics controls. A known membrane protein, gp28 (annotated holin) is predicted to have two TMDs (Fig. S2A), and an annotated minor tail protein, gp21, has no predicted hydrophobic domains, suggesting its cytoplasmic localization (Fig. S2B). These results are indicative of cytoplasmic localization for gp30 and membrane integration for gp31. We note that all *Actinobacteria* gp31 orthologues shown in Table 1 are predicted to have four TMDs by TMHMM (data not shown), while *Actinobacteria* gp30 orthologues are devoid of TMDs (data not shown).

10.1128/mSystems.00534-20.2 FIG S2 TMHMM (7, 8) prediction of transmembrane domains of Butters gp28 (annotated holin) and gp21 (annotated minor tail protein). Amino acids (aa) are plotted on the horizontal axis, and the blue, purple, and red lines indicate the probability of an amino acid being located inside, outside, or within the membrane of the cell, respectively. Protein gp28 is predicted to have two transmembrane domains, and gp21 is predicted to be a cytoplasmic protein. Download FIG S2, TIF file, 0.3 MB. Copyright © 2020 Mageeney et al. 2020 Mageeney et al. This content is distributed under the terms of the Creative Commons Attribution 4.0 International license.

I-TASSER (21) and Phyre (22) were used to further analyze gp30 and gp31 structures. Gp30 has weak homology with protein structures in the Protein Data Bank (PDB) and no distinguishing features (Fig. 1C). Butters gp31 is predicted to have 4 alpha-helices, which presumably, are membrane spanning in concordance with the TMHMM posterior probabilities for gp31 (Fig. 1D).

**FIG 2:**
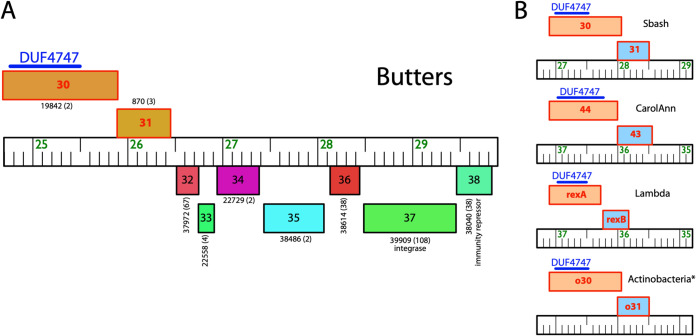
Genomic synteny of selected phage-encoded exclusive systems. (A) Central “variable region” of the Butters genome. The gene colors and numbers represent gene phamilies designated by the Phamerator database Actino_Draft version 353 (41); the number of phamily members is shown in parentheses. Rightward- and leftward-transcribed genes are shown above and below, respectively. The blue bar on top of gene *30* indicates the DUF4747 domain. Gene coloring is randomly produced by Phamerator. (B) Syntenic representation of two-component exclusion systems found in bacteriophages Sbash, CarolAnn, and Lambda. Butters genes *30* and *31* are compared to the Abi systems of Sbash, CarolAnn, and Lambda. *, Orthologous (o) genes with conserved synteny are also found in several *Actinobacteria* species, as detailed in Table 1. Genes (represented as boxes) are aligned to their genome (ruler) labeled with coordinates, except for the generic representation of genes in *Actinobacteria*. Gene coloring denotes similar functions for proteins encoded by these genes. The conserved DUF4747 domain is aligned on the putative cytoplasmic component of the exclusion system (blue bar). A nonsense mutation in the Rhodococcus baikonurensis gp30 orthologue (noted in Table 1) results in production of a truncated gp30 protein without an intact DUF4747 domain. Transcription is from left to right in all cases. The genomes of CarolAnn and Lambda have been reversed to aid comparison.

Gp30 and gp31 were also analyzed using HHpred to investigate their function (23, 24). HHpred analysis of gp30 yields a weak hit to the motif DUF4747 (probability, 69.48; E value, 140) (Fig. 2A). This DUF4747 domain is conserved in the cytoplasmic components of the Abi systems uncovered in coliphage Lambda (RexA) (25, 26), Mycobacteriophage Sbash (gp30) (15), and *Gordonia* phage CarolAnn (gp44) (16) (Fig. 2B). Lambda cytoplasmic RexA (when activated by a protein-DNA complex of the invading phage) binds to the membrane protein RexB (an ion channel), which depolarizes the membrane, resulting in loss of intracellular ATP, death of the bacterium, and abortion of infection (27). Similar mechanisms of action have been proposed for the Abi systems of Sbash (15) and CarolAnn (16). Remarkably, Butters gp31 and all the membrane components of these Abi systems have 4 transmembrane domains (Fig. 1 and Fig. S3). These structural similarities highlighted the possibility that Butters gp30 and gp31 may play roles in prophage-mediated defense and intimated possible functional similarities with the RexAB Abi system as well. Butters gp31 has weak homology to bacteriophage holins from *Enterobacter* phage P21 (probability, 58.8; E value, 25), *Haemophilus* phage HP1 (probability, 52.88; E value, 39), and pneumococcal phage Dp-1 (probability, 21.24; E value, 550) and to a bacteriophage holin family, superfamily II-like (probability, 64.23; E value, 26) (28). However, it is atypical for holin proteins to have more than two TMDs (29). Moreover, gene *31* is expressed in the Butters lysogenic cycle (14), rendering a holin function unlikely for gp31.

10.1128/mSystems.00534-20.3 FIG S3 TMHMM (7, 8) prediction of transmembrane domains of Sbash gp31, CarolAnn gp43, and Lambda RexB. Amino acids (aa) are plotted on the horizontal axis, and the blue, purple, and red lines indicate the probability of an amino acid being located inside, outside, or within the membrane of the cell, respectively. Posterior probabilities reveal four transmembrane domains for all proteins. Download FIG S3, TIF file, 0.8 MB. Copyright © 2020 Mageeney et al. 2020 Mageeney et al. This content is distributed under the terms of the Creative Commons Attribution 4.0 International license.

### Phage infection assays indicate that gp30 and gp31 are components of a prophage-mediated defense system against viral attack.

**FIG 3:**
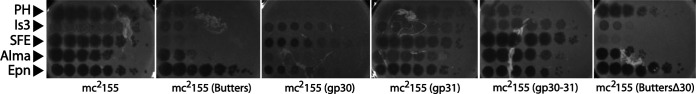
Plating efficiencies of heterotypic phages on M. smegmatis mc^2^155 strains expressing gp30, gp31, or gp30-31 [designated mc^2^155(gp30), mc^2^155(gp31), and mc^2^155(gp30-31), respectively]. Phages spotted are listed on the left as follows: PH (PurpleHaze), Is3 (Island3), SFE (ShrimpFriedEgg), Alma, Epn (Eponine). Phage lysates were serially diluted to 10^−7^ and spotted (3 μl each) onto a lawn of each bacterium plated with 1× top agar. ShrimpFriedEgg (cluster N) inhibition on mc^2^155(Butters) and mc^2^155(ButtersΔ*30*) is repressor mediated (14). mc^2^155(gp30) defends against PurpleHaze(A3) and Alma(A9) but not Island3(I1). gp30-mediated defense is attenuated in the presence of gp31. In agreement with previous results (14), Island3 and Alma show reduced plating efficiencies on mc^2^155(Butters). On both lysogen lawns, the absence of individual plaques in the dilution series for Island3 and ShrimpFriedEgg suggests that observed clearings are due to “killing from without” and not infection. At least three independent biological replicates for each strain, with *n* ≥ 3 technical replicates, were used for plating experiments. In no case did variation in EOPs between replicates exceed an order of magnitude.

Given the shared structural homology between Butters gp30 and gp31 and the Abi systems of coliphage Lambda, *Gordonia* phage CarolAnn, and mycobacteriophage Sbash (Fig. 2 and Fig. S3) coupled with the fact that all characterized cluster N mycobacteriophage prophage-mediated defenses have been mapped to genes within the central variable region of their genomes (14), we hypothesized that Butters genes *30* and *31* are involved in prophage-mediated defense. We tested this hypothesis using a phage infection assay. We spotted serial dilutions of a selected panel of heterotypic phages known to be inhibited by the Butters lysogen, Alma and Island3 (14; this study), and PurpleHaze (this study), on lawns of M. smegmatis mc^2^155 derivatives expressing Butters gene *30* alone, Butters gene *31* alone, and both Butters genes *30* and *31* represented as mc^2^155(gp30), mc^2^155(gp31), and mc^2^155(gp30-31), respectively (Fig. 3). All Butters genes were expressed from the integration-proficient vector pMH94 using the endogenous Butters promoter and ribosome binding site to drive gene expression (see details in Materials and Methods). Phage serial dilutions were also spotted on a Butters lysogen, mc^2^155(Butters), and a Butters lysogen variant with gene *30* deleted, mc^2^155(ButtersΔ*30*).

All phages efficiently infected an M. smegmatis mc^2^155 strain carrying the empty vector pMH94 (Fig. S4A). Eponine (subcluster K4) plated efficiently on all lawns while ShrimpFriedEgg (cluster N) was inhibited by the Butters lysogen, which expresses the Butters immunity repressor (Fig. 3 and Table S1). Heterotypic phages PurpleHaze (subcluster A3), Island3 (subcluster I1), and Alma (subcluster A9) had reduced efficiency of plating on an M. smegmatis mc^2^155(Butters) lawn (14; Fig. 3 and Table S1). Defense against heterotypic phages is independent of immunity repressor function (14); therefore, we predict that inhibition of PurpleHaze, Island3, and Alma infection would be mediated by other genes. M. smegmatis mc^2^155 strains expressing Butters gp30 alone completely abolished PurpleHaze infection and reduced infection of Alma by 4 orders of magnitude but had no apparent effect on Island3 infection (Fig. 3 and Table S1). These results delineate the presence of at least two distinct defense mechanisms encoded by the Butters prophage against heterotypic phages, one mediated by gp30 and the other, gp30 independent. Remarkably, while the strain expressing only gp31 had no inhibitory effect on all phages tested, coexpressing gp31 with gp30 attenuated the inhibitory effect gp30 had on PurpleHaze and completely abolished gp30 antagonism of Alma (Fig. 3 and Table S1). This establishes a functional interaction between gp30 and gp31.

10.1128/mSystems.00534-20.4 FIG S4 (A) Plating efficiencies of heterotypic phages. Related to Fig. 3. All phages were plated efficiently on the M. smegmatis mc^2^155 strain carrying the empty vector pMH94. (B) Butters prophage-mediated defense against Island3 is not repressor mediated. Island3 plates efficiently on a ShrimpFriedEgg lysogen [mc^2^155(ShrimpFriedEgg)] but is inhibited on a Butters lysogen [mc^2^155(Butters)]. Repressor-mediated immunity accounts for inhibition of ShrimpFriedEgg on both cluster N lysogens. At least three independent biological replicates for each strain, with *n* ≥ 3 technical replicates, were used for plating experiments. In no case did variation in EOPs between replicates exceed an order of magnitude. Download FIG S4, TIF file, 0.6 MB. Copyright © 2020 Mageeney et al. 2020 Mageeney et al. This content is distributed under the terms of the Creative Commons Attribution 4.0 International license.

10.1128/mSystems.00534-20.8 TABLE S1 Sensitivities of Mycobacterium smegmatis mc^2^155 strains to infection by mycobacteriophages. Numbers indicate the efficiencies of plating (EOP) of phages on Mycobacterium smegmatis mc^2^155 strains carrying part or all of the Butters genome compared to wild-type Mycobacterium smegmatis mc^2^155. EOPs ≥10^−3^ (shown in bold) are significant. *, the observed bacterial clearing is presumed to be “killing from without,” a phenomenon where at high phage concentrations, bacteria are killed due to the overwhelming number of phages adsorbing to its membrane. This contrasts with the conventional phage infection process where a single phage particle injects its DNA into a bacterium, makes more copies of the virion particle, and bursts the cell to release the progeny phages to start a new infection cycle. The absence of single plagues further out in the dilution series suggests that clearings represent “killing from without.” Download Table S1, DOCX file, 0.02 MB Copyright © 2020 Mageeney et al. 2020 Mageeney et al. This content is distributed under the terms of the Creative Commons Attribution 4.0 International license.

Next, we tested phages on mc^2^155(ButtersΔ*30*). For PurpleHaze, the absence of gene *30* resulted in near total recovery of infection (Fig. 3 and Table S1). Therefore, inhibition is almost exclusively dependent on the presence of Butters gp30. On the other hand, infection by Island3 is still inhibited, implicating a gp30-independent mechanism for defense against this phage. Island3 plates efficiently on another cluster N phage lysogen (mc^2^155[ShrimpFriedEgg]), demonstrating that defense against Island3 is not repressor mediated (Fig. S4B). Collectively, our data support the proposal that repressor-mediated immunity accounts for defense against homotypic phage infection, but not against heterotypic viral infection, and that multiple defense mechanisms against heterotypic viral attack are specified within the Butters genome.

### Microscopy reveals a functional link between gp30 and gp31.

To visually confirm the localization of gp30 and gp31 predicted by bioinformatics analyses (Fig. 1) and explore a possible physical interaction between gp30 and gp31, we performed fluorescence microscopy experiments. To minimize the possible effects of fluorescent probes in the function and cellular localization of our proteins of interest, we used the FlAsH system (Materials and Methods) to tag gp30 (gp30T) and gp31 (gp31T). M. smegmatis mc^2^155 expresses endogenous proteins with amino acid domains recognized by the FlAsH dye, thus limiting its specificity (Fig. S5). For this reason, and given the successful precedent of heterologous expression of mycobacterial and mycobacteriophage proteins in E. coli (30), we performed our imaging in wild-type strain K-12 MG1655.

10.1128/mSystems.00534-20.5 FIG S5 Snapshots for representative M. smegmatis wild-type cells using FlAsH dye. Wild-type M. smegmatis expresses a protein (or a number of proteins) that contains the TC tag motif, thus rendering this labeling method ineffective in this organism due to the loss of specificity for FlAsH. All scale bars denote 1 μm. Download FIG S5, TIF file, 0.3 MB. Copyright © 2020 Mageeney et al. 2020 Mageeney et al. This content is distributed under the terms of the Creative Commons Attribution 4.0 International license.

**FIG 4:**
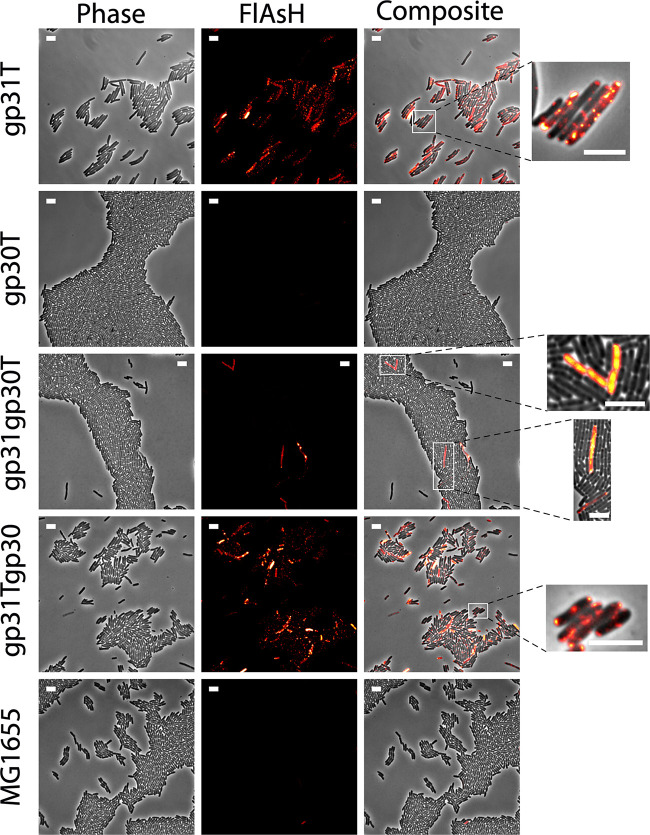
Snapshots of representative microscopy images of E. coli cells expressing gp31, gp30, and coexpressing gp30 and gp31 using the tetracysteine (FlAsH) tag detection system. Wild-type E. coli cells (MG1655) were used as the control. Proteins modified to include the FlAsH tag are indicated by a final letter T. All images have been normalized to the same fluorescence intensity scale. The white bar scale represents 5 μm in all cases. The zoomed images (right) highlight representative patterns of expression. Quantification of phenotypes and fluorescence average intensities are shown in Fig. S7.

While we observed cell-to-cell variability in the case of gp31, all MG1655(gp31T) cells showed a fluorescent signal located in evenly distributed clusters (Fig. 4). This pattern is compatible with predicted phage membrane protein integration as shown in previous studies (31) yet is different from membrane patterning for holins (32). On the other hand, MG1655(gp30T) cells did not reveal a significant signal for gp30 (Fig. 4). In order to check the efficiency of FlAsH labeling for Butters proteins with a predicted cytoplasmic localization, we performed control experiments using a strain expressing minor tail protein gp21, MG1655(gp21T). In that case, we found a consistent cytoplasmic signal (Fig. S6). Thus, while microscopy experiments showed the predicted localization of gp31, they were inconclusive with regard to gp30 localization.

10.1128/mSystems.00534-20.6 FIG S6 Snapshots of representative microscopy images of E. coli cells expressing Butters gp21 using the tetracysteine (FlAsH) tag detection system. The fluorescence intensity scale is as in Fig. 4. The white bar scale denotes 5 μm in all cases. The zoomed images (right) highlight representative patterns of expression. Quantification of the phenotype and fluorescence average intensity are shown in Fig. S7. Download FIG S6, TIF file, 0.9 MB. Copyright © 2020 Mageeney et al. 2020 Mageeney et al. This content is distributed under the terms of the Creative Commons Attribution 4.0 International license.

10.1128/mSystems.00534-20.7 FIG S7 Quantification of phenotypes (length and width of cells) and average cell fluorescence of microscopy images (Fig. 4 and Fig. S6). Download FIG S7, TIF file, 0.3 MB. Copyright © 2020 Mageeney et al. 2020 Mageeney et al. This content is distributed under the terms of the Creative Commons Attribution 4.0 International license.

To investigate if the proposed interaction suggested by the phage infection assay between gp30 and gp31 modifies the signal pattern, we developed strains coexpressing these proteins under the control of the same promoter. In one case, only gp30 was tagged to produce strain MG1655(gp31gp30T), whereas in the other strain, gp31 was tagged to create strain MG1655(gp31Tgp30). The signaling pattern for strain MG1655(gp31Tgp30) revealed intensity and distribution equivalent to the pattern observed when gp31 was expressed alone (Fig. 4). In the dual expressing strain where gp30 was tagged (MG1655[gp31gp30T]), only a few cells showed signal (Fig. 4 and Fig. S7). These cells consistently displayed two distinct patterns (Fig. 4). While some cells showed a pattern compatible to that expected for cytoplasmic localization, others showed a membrane pattern similar to that observed in strains where gp31 was tagged, MG1655(gp31T) and MG1655(gp31Tgp30).

As for the cell phenotype, we found that MG1655(gp31T) cells displayed an elongated phenotype, yet we did not observe filamentation (Fig. S7; 33). Our data also indicate that gp30-expressing cells have a phenotype compatible with that observed in wild-type cells (Fig. 4 and Fig. S7). Interestingly, in cells coexpressing genes *30* and *31*, the gp31-induced elongation phenotype was lessened (Fig. S7). Hence, the presence of gp30 diminishes the elongation phenotype observed when gp31 is expressed alone, supporting the proposal of a functional interaction between gp30 and gp31.

### Immunoprecipitation experiments hint at an interaction between gp30 and gp31.

**FIG 5:**
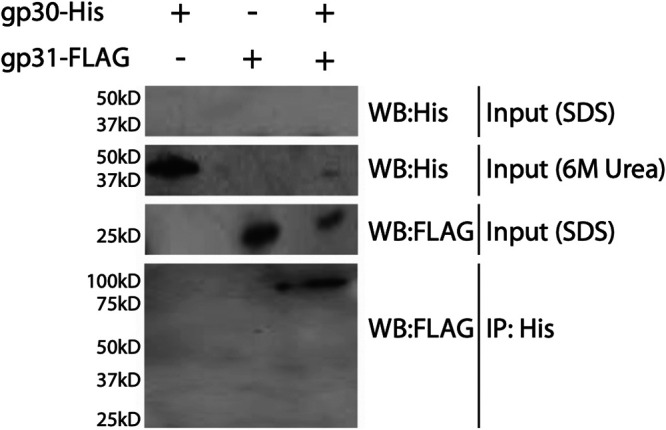
Butters gp30-His immunoprecipitation. Western analysis of BL21 E. coli cells expressing Butters gp30-His and Butters gp31FLAG alone or together. The input resuspended in 6 M urea shows the expected 40-kDa gp30-His protein in strains expressing gp30-His when probed with anti-His. The input resuspended in SDS lacks a 40-kDa moiety when probed with a His antibody, which may suggest the tag is masked and cannot be accessed by the antibody. Similarly, the input probed with a FLAG antibody shows gp31FLAG at 25 kDa in the gp31FLAG and dual strains. Following the His-IP, an ∼100-kDa band is visible when probed for FLAG, suggesting a stoichiometric relationship between gp30-His and gp31FLAG that is not 1:1.

The phage infection assay and microscopy experiments suggest a gp30-gp31 functional interaction. To explore the possibility of a physical interaction, we performed coimmunoprecipitation (co-IP) experiments using BL21 E. coli extracts from strains expressing FLAG-tagged gp31 or His-tagged gp30 or both. For Western blot analysis of the strain expressing gp30His alone, no immunoreactive signal at the predicted molecular mass of gp30His (∼40 kDa) was detected when the bacterial lysate, previously resuspended and boiled in SDS sample buffer, was probed with the anti-His antibody (Fig. 5). We therefore used 6-M urea for protein denaturation and observed an immunoreactive product at the expected molecular size of ∼40 kDa (Fig. 5). Following a His-IP using Ni^2+^-NTA magnetic beads and a lysate from the strain expressing both gp30His and gp31FLAG, our anti-FLAG probe detected a product at ∼100 kDa. Interestingly, this product is higher than the ∼61 kDa predicted for a complex of one molecule of gp30 (∼40 kDa) and one molecule of gp31 (∼21 kDa). Our inability to detect an immunoreactive signal for gp30His or for gp30His-gp31FLAG on probing with an anti-His antibody may be due to inaccessibility of the His-tag. Incomplete denaturation in SDS may not expose enough of the 6×His sequence/epitope for detection by the anti-His antibody, whereas Ni^2+^-NTA capture of His-tagged proteins can be successful with involvement of as few as two His residues (34). Overall, these results support the possibility of a physical interaction between gp30 and gp31.

## DISCUSSION

### Identification of *Mycobacterium* phage Butters transmembrane proteins gp31 and gp30 as components of a host antiviral defense system.

Numerous bacterial defense systems that protect against bacteriophage infection at multiple stages in the phage infection cycle have been described (reviewed in reference 35), with additional systems likely to be uncovered as comparative bacterial genomics continues to expand. It is important to note that Butters was isolated from a soil sample. Characterizing the defense mechanisms of Butters and other soil phages will be crucial to understand undiscovered biological interactions between microbes and their phages within soil environments and the impact on soil ecology. Equally important within microbial communities are bacteriophage counterattack mechanisms that subvert bacterial defense efforts (reviewed in reference 36). For temperate phages, mutually beneficial host-phage interactions have evolved to support efficient propagation of both bacteria and phages and to maintain lysogeny. Expression of prophage genes contributes to a profile of potentially unique capabilities within the bacterial host, including new functions that affect numerous aspects of bacterial physiology and metabolism and, in the context of the work described here, new capabilities that specify defense mechanisms that alter the phage resistance phenotype of the host.

The recent discovery of genes within cluster N mycobacteriophage genomes that function as part of host defense mechanisms against heterotypic viral attack when expressed from the prophage in a cluster N lysogen has broadened our understanding of the diversity of antiphage defense systems and coevolving counterattack viral systems (14). These prophage-mediated defense systems are highly specific, even differentiating between different phages within the same subcluster (14). At least five different defense mechanisms were uncovered, including a single-subunit restriction system in cluster N phage Panchino, a heterotypic exclusion system in cluster N phage Charlie, and a predicted (p)ppGpp synthetase in cluster N phage Phrann, which inhibits lytic phage growth and facilitates efficient lysogeny (14). In each case described, relevant phage genes mediating defense are positioned within a centrally located variable region of the phage genome and are highly expressed in RNAseq profiles from cluster N lysogens (14). For mycobacteriophage Butters, genes involved in defense had not previously been identified experimentally, nor had any experimental validation related to protein localization been completed. Genes *30* and *31* were originally of interest because of their novel representation as orphams among all known mycobacteriophage genes analyzed at the beginning of these studies; the presence of orthologous genes in several *Actinobacteria*, including strains of clinical relevance, further elevates interest in uncovering the molecular roles for these genes. Insights about gp30 and gp31 localization were revealed using computational tools (TMHMM, I-TASSER, Phyre) to predict membrane domains. The existence of a conserved protein domain identified by HHpred informed predictions about protein functions.

We coupled bioinformatics analyses with fluorescence imaging of tagged proteins in MG1655 E. coli and plating efficiencies of heterotypic phages on M. smegmatis mc^2^155 strains expressing Butters proteins gp30 and gp31 to provide experimental validation for the proposal that gp30 and gp31 are components of a prophage-mediated antiviral system expressed within a Butters lysogen. Computational predictions that Butters gp31 is a membrane protein are supported by fluorescence imaging of MG1655 E. coli cells expressing Butters gp31. In this case, gp31 is found in association with the E. coli membrane and, by inference, we conclude that Butters gp31 would likewise be incorporated into the membrane of an M. smegmatis host as well. As for Butters gp30, microscopy experiments using strains expressing gp30 alone were not conclusive with respect to its subcellular localization since cells only displayed a signal with levels slightly above background (Fig. S7). Still, when gp30 was coexpressed with gp31 our data pointed toward an interaction between gp30 and gp31. On the one hand, we observed a phenotypic change (the gp31-induced cell elongation was lessened), demonstrating a functional interaction. On the other hand, we systematically observed some cells with a gp30 expression pattern compatible with either a membrane localization or a cytoplasmic localization. Taken together, these results and evidence from immunoprecipitation assays hint at a physical interaction between gp30 and gp31 and are suggestive of conformational remodeling.

### Model for a prophage-encoded exclusion system to prevent heterotypic phage infection.

Several mechanisms have been uncovered to account for resistance or immunity from viral attack within bacterial lysogens. Repressor-mediated immunity accounts for the ability of an immunity repressor (encoded by a prophage) to inhibit the lytic cycle and superinfection by homotypic phages harboring a similar immunity system. In this study, repressor-mediated immunity accounts for inhibition of infection by homotypic cluster N phage ShrimpFriedEgg on Butters and ButtersΔ*30* lysogen lawns. Superinfection exclusion (Sie) prevents viral attack from heterotypic phages with dissimilar immunity systems by likely blocking DNA entry into host cells, which results in resistance to infection by certain phages. Unlike repressor-mediated and Sie systems that block phage superinfection, Abi systems counter phage attack but lead to host cell death. These systems may target any stage of the phage infection cycle, including DNA replication, transcriptional activation, or translation to eradicate the phage threat but, in doing so, also abolish the life of the host cell as well (27).

A widely studied Abi system is the Rex system, a two-component protection system of the proteins RexA and RexB, encoded by the Lambda prophage in an E. coli lysogen to prevent lytic phage superinfection (reviewed in reference 27). In this system, inactive RexA is activated in the cytoplasm through interactions with an invading phage DNA-protein complex following phage adsorption and DNA injection. Two activated RexA proteins bind the transmembrane protein RexB, which functions as an ion channel. Influx of ions disrupts membrane potential, leading to host cell death, and ultimately, quenches phage infection. Interestingly, an additional function proposed for RexB (37) is to prevent Lambda phage self-exclusion following induction of a lysogen (38). Changes in the ratio of RexA and RexB are proposed to impact superinfection exclusion (39).

The low degree of structural similarity between RexA and Butters gp30 (shown by the DUF4747 domain) would not typically be used to assign a functional prediction due to the low probability and high E score. However, the presence of this stretch of homology (also conserved in cytoplasmic components of analogous Abi systems described in *Gordonia* phage CarolAnn and mycobacteriophage Sbash) may provide clues for how gp30 may function in conjunction with gp31. Butters gp31, RexB, and the membrane components of CarolAnn and Sbash Abi systems are all 4-pass transmembrane proteins. Additionally, the established stoichiometry between the two components of the Abi systems described includes two molecules of the RexA-like protein binding to one molecule of the RexB-like protein. Although not detected in a reciprocal co-IP experiment (FLAG co-IP, data not shown), the ∼100-kDa product for the proposed Butters gp30/gp31 complex observed in our His-co-IP is consistent with stoichiometry for RexA/B.

Although several structural similarities between Butters gp30/gp31 and the Abi systems described may suggest that Butters gp30/gp31 share some functional attributes with these systems, substantial differences exist based on our experimental analyses. First, Butters gp30 is sufficient to abolish infection by PurpleHaze and Alma. This contrasts sharply with the previously described Abi systems, where the cytoplasmic component is insufficient to inhibit infection. For the recently described two-component systems in Sbash and CarolAnn, both genes are required to confer the defense phenotype (15, 16). Second, in the previously described Abi systems, the cytoplasmic component requires activation from components of the invading phage prior to binding to the membrane-bound component. However, even in the absence of a “sensing” phage component, our microscopy and co-IP data suggest a functional link and potential physical interaction, respectively, between Butters gp30 and gp31. Our immunity experiments show that the Butters lysogen defends its host against infection by the heterotypic phages PurpleHaze, Island3, and Alma (Fig. 3). We note that the cluster N Rubeelu prophage, which differs from Butters by 24 single nucleotide polymorphisms, shows similar immunity dynamics with respect to PurpleHaze and Island3 (data not shown). Our strategy to construct M. smegmatis strains that individually express gp30 or gp31 or both allowed us to evaluate the contribution of each gene to the mechanism of antiviral defense displayed in the Butters lysogen. Our immunity data show that gp31 alone has no inhibitory effect on any phages tested, but Butters gp30 strongly inhibits infection by PurpleHaze and Alma (Fig. 3). This inhibition is attenuated when gp30 is expressed along with gp31. The Butters gp30/31 complex may harbor some inhibitory effect for PurpleHaze but not Alma. Collectively, these distinguishing features for the Butters defense system provide new insights into systems that are reminiscent of RexA/B-like systems but, importantly, highlight likely mechanistic differences.

**FIG 6:**
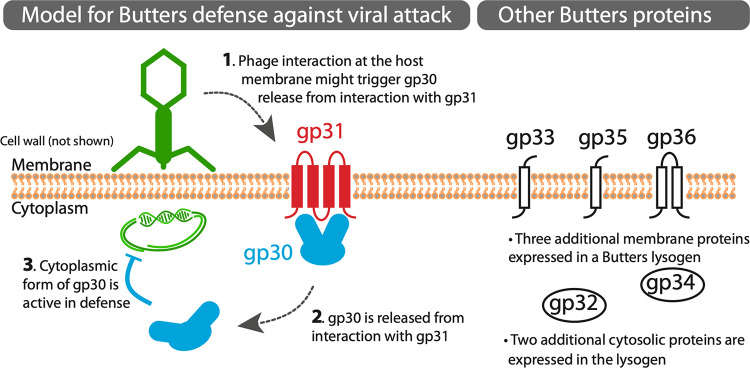
Model for Butters defense against viral attack. Mycobacteriophage Butters gp30 and gp31 are proposed to interact at the membrane. Numbers in cartoon arrows indicate the sequence of events. (1) gp30 release from gp31 is mediated by an unknown mechanism and may be triggered by phage interaction or gp31 interactions with other phage or host proteins. (2) When gp30 is released from interacting with gp31 at the membrane, it is liberated into the cytosol. (3) The cytoplasmic form of gp30 may facilitate host defense against select viral infections. Host defense may proceed following phage adsorption and subsequent DNA injection. Dashed arrows correspond to unconfirmed hypotheses. The Butters proteins shown are expressed from the variable region (between the lysis and immunity cassettes). The complete prophage expression profile is described (14). Three additional membrane proteins (gp33, gp35, gp36) and two additional cytoplasmic proteins (gp32, gp34) are expressed from the “variable region” of Butters (right panel). The roles of these five additional proteins in prophage-mediated defense are unknown but may include additional defense mechanisms against other heterotypic phages. Some phages escape all mechanisms of defense mounted within a Butters lysogen.

We therefore propose a two-component model whereby gp30 and gp31 form a complex at the membrane in the absence of heterotypic phage infection. Gp30 may be released from the membrane complex when the host is challenged by phage adsorption and DNA injection (e.g., from PurpleHaze), allowing gp30 to exert its antiviral effect as a cytoplasmic component (Fig. 6). Preliminary adsorption assays suggest that PurpleHaze adsorption is not blocked, since adsorption efficiencies are equivalent for wild-type M. smegmatis and recombinant strains expressing Butters genes (C. M. Mageeney, unpublished data). Whether or not the DUF4747 domain of gp30 binds a DNA-protein complex is unknown. While gp30 is clearly the main antagonizing protein and could, alternatively, be proposed as a single-component, antiphage system, we propose that a minimal role for gp31 in modulating gp30 function must be considered based on our plating efficiency data for both PurpleHaze and Alma infections. For PurpleHaze, it is equally likely that inhibition is mediated by the undissociated gp30/gp31 complex since the efficiency of plating on mc^2^155(gp30-31) and on the Butters lysogen is equivalent. It remains unknown whether or not the Butters gp30/31 defense system targets DNA or specific proteins of the invading phage, but the targets of Sbash gp30/31 (Crossroads gp132/141) and CarolAnn gp43/44 (Kita gp53) have no homologues in either PurpleHaze or Alma.

The presence of gp30 and gp31 orthologues within Actinobacteria and conservation of their genetic syntenic framework (Table 1) (as is found in Butters) bolsters the argument that these proteins function as a two-component system. Utilization of these proteins in defense mechanisms against viral attack could be widespread among bacterial isolates, including clinically relevant strains. Understanding the roles of these two proteins as well as the mechanisms of action could allow for advances in therapeutic or industrial applications of phages.

Interestingly, defense against cluster I1 phage Island3 must proceed by an alternative mechanism(s) since the M. smegmatis strain expressing gp30 alone or gp30 and gp31 combined provides no protection from Island3, yet the Butters lysogen provides antiviral protection against this phage. Defense against Island3 is not repressor mediated, as demonstrated by the inability of the ShrimpFriedEgg repressor to block Island3 infection (Fig. S4B). Moreover, the ButtersΔ*30* strain marginally defends against PurpleHaze and Alma, further suggesting the presence of additional defenses independent of the actions of gp30. Our results do not clarify whether the same gp30-independent defense mechanism is responsible. Within the variable region of the Butters genome, at least five other genes (*32* to *36*, not including the repressor [gene *38*]) are also expressed from the prophage genome (14). These genes may also promote antiviral defense. Thus, the Butters prophage contributes to an array of different prophage-induced defense systems within the host.

Overall, several features of the model are amenable to biochemical analyses using our M. smegmatis strains. Analysis of defense escape mutants will no doubt be useful in deciphering the mechanism by which heterotypic phages are excluded from infection of a Butters lysogen. Altogether, our work may reveal a novel mechanism of virally encoded defense systems that protect the bacterial host against attack by heterotypic phages. These studies open the door for understanding defense mechanisms within pathogenic bacteria that may interfere with development of biocontrol strategies against bacterial infections.

## MATERIALS AND METHODS

### Bioinformatics analysis.

Transmembrane regions were predicted for each protein-coding gene by submitting protein sequences to TMHMM (19, 20). Structural predictions were made for Butters gp30 and gp31 using I-TASSER (21) and Phyre (22). Five models were predicted for Butters gp30, with the highest C-score being –4.00. The highest score alignment with protein structures in the PDB identify hydroxycinnamoyl-coenzyme A (CoA):shikimate hydroxycinnamoyl transferase from Sorghum bicolor (PDB 4ke4A; template modeling [TM] score, 0.881). Five models were predicted for Butters gp31, with the highest C-score being –3.65. The highest score alignment with protein structures in the PDB identify Niemann-Pick C1 protein from Homo sapiens (PDB 3jd8A3; TM score, 0.723). Phyre predicts similar structures with very low homology to known proteins for both gp30 and gp31. Amino acid sequences for gp30 and gp31 were submitted to HHpred (23, 24) to search for proteins with similar amino acids and/or domains using the NCBI Conserved Domains Database version 3.18 (default settings).

### Phage isolation, propagation, and genomic analysis.

Phages (GenBank accession numbers KC576783 [Butters], KY965063 [PurpleHaze], HM152765 [Island3], MK524528 [ShrimpFriedEgg], JN699005 [Alma], and MN945904 [Eponine]) were isolated and grown on Mycobacterium smegmatis mc^2^155 as previously described (40). PurpleHaze, Island3, and Alma lysates were obtained from the Hatfull lab (University of Pittsburgh). The genomic sequence for the Island3 strain used in this study differs from that of the wild type with a 257-bp deletion (coordinates 43307 to 43563) and a C2656T single nucleotide polymorphism (SNP). Phage lysates (titers, ≥1 × 10^9^ PFU/ml), diluted with phage buffer (0.01 M Tris, pH 7.5, 0.01 M MgSO_4_, 0.068 M NaCl, and 1 mM CaCl_2_), were used for immunity testing and PCR. Phamerator Actino_Draft version 353 (41) was used for comparative genomic analysis and genome map representation.

### Construction of Butters Δ gene *30* phage mutant.

The Δ*30* phage mutant was constructed using a modification of the bacteriophage recombineering of electroporated DNA (BRED) approach as previously described (14). Four primers, along with Butters genomic DNA (purified by phenol-chloroform extraction) and Platinum high-fidelity PCR supermix (Invitrogen), were used in a three-step PCR strategy to generate a recombination substrate (1,318 bp) for gene deletion. The genomic coordinates for Butters gene *30* are 24688 to 25899. In PCR1, primers 1 (coordinates 24200 to 24223) and 3 (reverse coordinates 24685 to 24661 fused to coordinates 25879 to 25870) were used to generate an ∼490-bp amplicon. In PCR2, primers 2 (coordinates, 24684 to 24697 merged with coordinates 25870 to 25899) and 4 (reverse coordinates 26700 to 26677) in PCR generated an ∼840-bp amplicon. Primers 1 and 4 along with equal molar amounts of PCR1 and PCR2 amplicons (to create a PCR3 template with ∼25 nucleotides of complementarity from PCR1 and PCR2 products) were used to generate the recombination substrate (∼1,318 bp) with gene *30* deleted. The PCR-generated substrate was used for BRED after agarose gel purification, PCR cleanup (Promega), and quantification. Purified substrate (100 ng) and 150 ng of Butters genomic DNA were coelectroporated into recombineering-efficient strain M. smegmatis mc^2^155 carrying plasmid pJV53. Cell recovery, plating, PCR screening, plaque purification, and amplification were conducted as previously described (14). Mutant phage genomic DNA was purified and sequenced at the Pittsburgh Bacteriophage Institute as previously described (42). The mutant gene *30* allele contains intact 5′ flanking sequences upstream of the translation start of gene *30* fused to 30 bp from the very 3′ end of gene *30*, removing 1,182 bp of gene *30* (spanning coordinates 24688 to 25870). The remaining mutant phage genomic sequence is identical to Butters (NCBI RefSeq NC_021061) except for a T to A SNP (at coordinate 25884). Primers for BRED and mutant plaque screening are shown in Table S3.

### Construction and characterization of lysogenic and recombinant M. smegmatis strains.

Butters and ButtersΔ*30* lysogens were created as previously described (14) and stably maintained with no evidence of loss of lysogeny.

Recombinant strains to express Butters genes *30*, *31*, and *30_31* were created as follows. All primers used in this study are shown in Table S3. All genes were cloned into the XbaI site of integration-proficient, kanamycin (KAN)-resistant, and ampicillin (AMP)-resistant vector pMH94 (43) using conventional restriction enzyme/ligation methods. PCR primers (Integrated DNA Technologies) were designed with a 5′ end XbaI site. Phage genes were amplified from Butters genomic DNA by PCR using Q5 high-fidelity DNA polymerase (New England Biolabs). All PCR products contained the entire 179 bp between gene *29* and gene *30* (containing the endogenous promoter and ribosome binding site [RBS]) to drive expression of genes *30* to *31.* PCR products were digested with XbaI overnight (O/N), purified by gel extraction, and ligated into XbaI-digested pMH94 using T4 DNA ligase (New England Biolabs) at 16°C O/N. Chemically competent E. coli were transformed and plated onto Kan/Amp plates, and colonies were screened by PCR with primers flanking the cloning site. Recombinant plasmids were verified by sequencing (Genscript).

Electrocompetent M. smegmatis mc^2^155 cells were prepared and transformed with recombinant pMH94 plasmids as previously described (44). After recovery, cells were plated on selective medium containing Luria broth agar with 50 μg/ml kanamycin. Strains were grown in 7H9 medium enriched with albumin (5%) and dextrose (2%) (AD supplement), 1 mM CaCl_2_, 50 μg/ml kanamycin, 50 μg/ml carbenicillin (CB), and 10 μg/ml cycloheximide (CHX) for 5 days at 37°C.

### Construction of pMH94_gp31.

The three-step PCR method briefly described above was used to generate a DNA segment containing the putative endogenous phage promoter and RBS and Butters gene *31.* All primers are listed in Table S3. Primers A and C were used to generate PCR_1, consisting of an XbaI site, all 179 bp of the intergenic region upstream of gene *30* and the first 19 bp of gene *31.* Primers B and D were used to produce PCR_2 consisting of the last 20 bp of the intergenic region upstream of gene *30*, the entirety of gene *31*, 42 bp downstream of gene *31*, and an XbaI site. PCR_1 and PCR_2 share a 39-bp overlap. PCR products were gel purified, and 20 ng of each was used as the template for the final PCR_3 using primers A and D to produce the gene *31* segment with the endogenous phage promoter and RBS. After gel purification, the PCR product was cloned into the XbaI site of pMH94 as described previously.

### Plating efficiency assays.

Lawns of M. smegmatis strains containing pMH94 recombinant plasmids or lysogens were made by plating 250 μl of the M. smegmatis strains with 3.5 ml of top agar on an LB agar plate (CHX/CB). Phage lysates were serially diluted to 10^−7^ and spotted (3 μl each) onto the M. smegmatis lawns of interest. Plates were incubated for 48 h at 37°C. Phage growth was assessed at 24 and 48 h, and efficiency of plating (EOP) was recorded after 48 h. EOP is calculated by first calculating the phage titer on each strain and then comparing the titers. Titer (plaque-forming units/ml) = (number of plaques/μl of phage spotted) · 1,000 μl/ml · inverse dilution. EOP = titer on experimental strain/titer on M. smegmatis mc^2^155.

### Plasmids for imaging strains.

All plasmids express one or two proteins of interest under the control of an inducible combinatorial promoter, P_lac/ara-1_ (45), tightly regulated by arabinose and isopropyl β-d-1-thiogalactopyranoside (IPTG). Dual strains coexpress gp31 and gp30, each with its own RBS. Plasmids were transformed into K-12 MG1655 E. coli cells. All strains used for imaging have the MG1655 genetic background (Table S2), except where we assessed FlAsH dye specificity in M. smegmatis (Fig. S5).

10.1128/mSystems.00534-20.9 TABLE S2 List of plasmids generated and bacterial strains used in this study. All plasmids that do not have a reference listed were generated for this study. The bacterial strain used for expression of each plasmid in this study are listed; plasmids with N/A were not expressed but were used for plasmid creation. Download Table S2, DOCX file, 0.02 MB. Copyright © 2020 Mageeney et al. 2020 Mageeney et al. This content is distributed under the terms of the Creative Commons Attribution 4.0 International license.

10.1128/mSystems.00534-20.10 TABLE S3 Oligos used in this study. T at the end of the protein name in the construct denotes the insertion of the tetracysteine tag motif in the C terminal of the protein. Download Table S3, DOCX file, 0.02 MB. Copyright © 2020 Mageeney et al. 2020 Mageeney et al. This content is distributed under the terms of the Creative Commons Attribution 4.0 International license.

E. coli SIG10 electrocompetent cells (Sigma-Aldrich, Saint Louis, ML) were used to clone plasmids using a combination of standard molecular cloning techniques and Gibson Assembly (master mix from New England Biolabs, Ipswich, MA). The plasmid pJS167 (46) was digested with EcoRI, and the desired region was amplified with primers F_pJS167EcoRI and R_pJS167EcoRI (Table S2) to create the ColE1 plasmid backbone. Posteriorly, constructs containing the gene(s) of interest (with or without the tetracysteine tag modification) were amplified from a Butters high-titer lysate using the corresponding primers detailed in Table S2 and cloned into the backbone using Gibson assembly. All plasmids were verified by sequencing.

### Microscopy/live-cell imaging.

To avoid expression of nonfunctional transmembrane proteins or artifacts during *in vivo* imaging due to fusion of the target protein to a “bulky'” fluorescent probe (e.g., green fluorescent protein [GFP]; 47), we used a biarsenical dye. This is a membrane-permeable dye that binds with high specificity to a small tetracysteine (TC) tag motif of six amino acids (Cys-Cys-Pro-Gly-Cys-Cys; 585 Da) included in the target protein sequence (48–50). We used the FlAsH green fluorophore (508/528 nm excitation/emission; Thermo Fisher Scientific).

To prepare the cells for microscopy, strains were grown O/N at 37°C with shaking in Luria broth (Miller’s modification, LB) with the corresponding antibiotic (ColEI, 50 μg/ml KAN) in a cell culture volume of 10 ml. Overnight cultures were diluted 1:100 into 5 ml of fresh A minimal medium (for 40 ml A minimal medium, 28 ml double-distilled water [ddH_2_O], 40 μl MgSO_4_.7H_2_O [1 M], 100 μl glycerol [80%], 4 ml CasaAa [1%], 800 μl glucose [20% wt/vol; [glucose]f = 0.4% wt/vol], and 8 ml A salts [for 5× A salts, 1 g ammonium sulfate [(NH_4_)_2_SO_4_], 4.5 g potassium dihydrogen phosphate (KH_2_PO_4_), 10.5 g potassium phosphate dibasic (K_2_HPO_4_), 0.5 g sodium citrate, 2H_2_O, and 200 ml sterile ddH_2_O (salts filter sterilized only)]) with inducers (ColEI, 0.7% arabinose; 2 mM IPTG) and cultured for 3 h at 37°C with shaking (for a final volume of 5 ml, 50 μl of the O/N culture was used). Then, 1 ml of cell culture was centrifuged (1,500 × *g* for 10 min) and resuspended in 500 μl of fresh A minimal medium with inducers. FlAsH labeling was conducted as follows: 1.25 μl of dye stock (2 mM), for a final concentration of 5 μM, was added, followed by a gentle vortex and incubation for 45 min at room temperature (RT) in the dark. Excess dye was removed by centrifugation at 1,500 × *g* for 10 min and resuspension in 1 ml of washing buffer. To reach a final concentration of 100 μM buffer per sample, 8 μl of bronchoalveolar lavage (BAL) buffer stock (100×, 25 mM) was added to 2 ml of A minimal medium with inducers. Cell cultures were incubated with washing buffer for 5 min at RT, and then this was repeated twice to remove any unbound or weakly bound tag. Cells were pelleted by centrifugation and resuspended in 500 μl of A minimal medium with inducers.

Cells (2 μl) were loaded on 2% agarose pads prepared as follows. A minimal medium (10 ml) and 0.2 g low-melting agarose were dissolved homogeneously by heating. After cooling, inducers were added, and the solution was filtered with 0.2-μm pore size membranes. The agarose solution was poured onto a coverslip and covered with another coverslip and allowed to dry for ∼1 h before microscopy.

Snapshots were taken at 37°C using an inverted microscope (Leica DMi8) equipped with a ×100/1.40 NA oil objective (HC PL APO, Leica), Kohler illumination conditions, a CMOS camera (Hamamatsu ORCA-Flash4.0 V2), and a GFP filter (excitation, 470/40 nm; emission, 525/50 nm). Excitation was performed using a light-emitting diode (LED) lamp (Lumencore SOLA SE), ensuring that the light intensity remained constant during experiments. The time exposure for phase contrast acquisition was set between 5 and 10 ms, and for FlAsH excitation, at 80 to 85 ms in all cases.

### Image processing and quantification.

Data analysis for snapshots was performed with Fiji (ImageJ). Background (fluorescence channel) was subtracted using the sliding paraboloid feature (50-pixel radius). The minimum level of background fluorescence was determined using strain MG1655(gp31T), and that set the cutoff signal level for characterizing the fluorescence signal in TC-tag-labeled strains. Images were processed using the Oufti toolbox (https://oufti.org; 33) to segment cells and perform an initial quantification of phenotypes (length/width of cells) and fluorescence levels. Manual correction of defective segmentation was implemented. We used the “spot detection” module in the Oufti software to detect and quantify clusters (gp31T and gp31Tgp30 strains). We developed custom-made Matlab code (Data_Processing.m) to process data sets and obtain final statistics about cell length, width, mean fluorescent intensity, and spot/cluster density for gp31T and gp31Tgp30.

### Coimmunoprecipitation assay.

Two plasmids were constructed. pEXP5/Buttersgp30His was constructed according to the manufacturer’s instructions for pEXP5-CT-TOPO cloning (Invitrogen). pEXP5/Kan/Buttersgp31FLAG was constructed by PCR amplification of Butters gene *31* with a FLAG tag and RBS. A pEXP5/kanamycin plasmid was created by replacing the AMP gene (by restriction endonuclease excision) with a KAN gene from pENTR-d-TOPO (Invitrogen) generated through PCR amplification. The KAN PCR amplicon with compatible ends was ligated into the plasmid backbone using T4 DNA ligase (Promega). The resultant pEXP5/Kan vector was linearized using XbaI, and Butters gp31FLAG was ligated into the plasmid for transformation into chemically competent BL21 cells. For expression, cells were grown O/N, diluted back to an optical density at 600 nm (OD_600_) of 0.04, and induced with 1 mM IPTG to grow for 5 h. Cells were harvested by centrifugation and lysed by sonication in 1× phosphate-buffered saline (PBS). Whole-cell lysates were added to His beads (Thermo Scientific HisPur Ni-NTA magnetic beads; PI88831) and incubated O/N at 4°C. Beads were washed with modified wash buffer (PBS, 50 mM imidazol pH 8), resuspended in SDS-sample buffer containing β-mercaptoethanol, and incubated at 95°C for 3 min prior to Western analysis. Whole-cell extract inputs were prepared by trichloroacetic acid (TCA) precipitation followed by either resuspension in 2 × SDS-sample buffer with β-mercaptoethanol or in 30 μl of 6 M urea and 2 × SDS-sample buffer with β-mercaptoethanol. Inputs were boiled for 10 min.

### Western analysis and antibodies.

Proteins were separated by SDS-PAGE and electrotransferred onto Westran-S PVDF membrane (Whatman number 10413096) as previously described (51). Primary antibodies (anti-FLAG [Sigma; F3165], anti-His [Cell Signaling Technologies, Danver, MA; 2366S]) were used at 1:1,000. Secondary horseradish peroxidase (HRP) conjugated goat anti-mouse IgG antibodies (Promega, Madison, WI; W4021) were used at 1:50,000.

### Data availability.

The genome sequences of all phages used in this study are available at https://phagesdb.org. GenBank accession numbers are provided in Materials and Methods. Sequences for constructs in this study are available by request. Microscopy images and the custom-made Matlab code to process data output from Oufti software (Data_Processing.m) are available by request.
