# Supplementary material for: Mycobacterium Phage Butters-Encoded Proteins Contribute to Host Defense against Viral Attack
Source: mSystems. 2020 Oct 6;5(5):e00534-20. doi: 10.1128/mSystems.00534-20 (PMC7542560; doi:10.1128/mSystems.00534-20)
Supplement: TABLE S1 [file mSystems.00534-20-st001.docx]

**Table S1:**

| **Strain**  **Phage** | mc^2^155  (Butters) | mc^2^155  (gp30) | mc^2^155  (gp31) | mc^2^155  (gp30-31) | mc^2^155  (Butters∆*30*) | mc^2^155  (pMH94) |
| --- | --- | --- | --- | --- | --- | --- |
| PurpleHaze | **10^-5^** | **0** | 1 | **10^-4^** | 10^-2^ | 1 |
| Island3 | **10^-4^*** | 10^-1^ | 1 | 10^-1^ | **10^-5^*** | 1 |
| ShrimpFriedEgg | **10^-5^*** | 10^-1^ | 1 | 1 | **10^-5^*** | 1 |
| Alma | **10^-3^** | **10^-4^** | 1 | 1 | 10^-2^ | 1 |
| Eponine | 1 | 10^-1^ | 1 | 1 | 1 | 1 |

.
