# Supplementary material for: Mycobacterium Phage Butters-Encoded Proteins Contribute to Host Defense against Viral Attack
Source: mSystems. 2020 Oct 6;5(5):e00534-20. doi: 10.1128/mSystems.00534-20 (PMC7542560; doi:10.1128/mSystems.00534-20)
Supplement: TABLE S2 [file mSystems.00534-20-st002.docx]

**Table S2:**

| **Plasmid name** | **Description/**  **Reference** | **Bacterial host** | **Strain name and experimental use** |
| --- | --- | --- | --- |
| pMH94 | capable of site-specific integration into chromosome of *M. smegmatis* mc^2^155 using the *int* gene of mycobacteriophage L5 and has KanR (ref. 1) | N/A | N/A |
| pMH94 | empty vector (ref. 1, 2) | *M. smegmatis* mc^2^155 | mc^2^155(pMH94); used in plating efficiency assay |
| pMH94_Buttersgp30 | Butters gene *30*  cloned into the Xba1 site of pMH94 | *M. smegmatis* mc^2^155 | mc^2^155(gp30); used in plating efficiency assay |
| pMH94_Buttersgp31 | Butters gene *31*  cloned into the Xba1 site of pMH94 | *M. smegmatis* mc^2^155 | mc^2^155(gp31); used in plating efficiency assay |
| pMH94_Buttersgp30-31 | Butters gene *30* and gene *31*  cloned into the Xba1 site of pMH94 | *M. smegmatis* mc^2^155 | mc^2^155(gp30-31); used in plating efficiency assay |
| N/A | Butters lysogen with gene *30* deleted | *M. smegmatis* mc^2^155 | mc^2^155(ButtersΔ*30*); used in plating efficiency assay |
| pEXP5-CT TOPO | Invitrogen V96006 (ref. 3) | N/A | N/A |
| pENTR/D-TOPO | Invitrogen K240020 | N/A | N/A |
| pEXP5/Kan | pEXP5-CT-TOPO with ampicillin gene removed and kanamycin gene added | N/A | N/A |
| pEXP5/Buttersgp30His | pEXP5-CT-TOPO with Butters gene *30* (24688-25896) with 3’- His tag | *E. coli* BL21 | N/A; used in Co-IP experiments |
| pEXP5/Kan/  Buttersgp31FLAG | pEXP5/Kan with Butters gene *31* (25892-26442) with 3’-FLAG tag | *E. coli* BL21 | N/A; used in Co-IP experiments |
| N/A | (ref. 4) | *M. smegmatis* mc^2^155 | mc^2^155; used in plating efficiency assays and imaging studies |
| ColE1/backbone | ColE1 plasmid-backbone containing Para/lac promoter controlling the expression of gene of interest (ref. 5) | N/A | N/A |
| ColE1/gp21T | ColE1/backbone containing gene *21* with the 3’- tetracysteine (TC) tag | *E. coli* MG1655 | MG1655(gp21T); used in imaging studies |
| ColE1/gp31 | ColE1/backbone containing gene *31* (without the TC tag) | *E. coli* MG1655 | MG1655(gp31); used in imaging studies |
| ColE1/gp31T | ColE1/backbone containing gene *31* with the 3’- TC tag | *E. coli* MG1655 | MG1655(gp31T); used in imaging studies |
| ColE1/gp30T | ColE1/backbone containing gene *30* with the 3’- TC tag | *E. coli* MG1655 | MG1655(gp30T); used in imaging studies |
| ColE1/gp31_30T | ColE1/backbone containing gene *31* (without the 3’- TC tag) and gene *30* with the 3’-TC tag | *E. coli* MG1655 | MG1655(gp31_30T); used in imaging studies |
| ColE1/gp31T_30 | ColE1/backbone containing gene *31* with the 3’-TC tag and gene *30* (without the 3’-TC tag) | *E. coli* MG1655 | MG1655(gp31T_30); used in imaging studies |
