# Supplementary material for: Mycobacterium Phage Butters-Encoded Proteins Contribute to Host Defense against Viral Attack
Source: mSystems. 2020 Oct 6;5(5):e00534-20. doi: 10.1128/mSystems.00534-20 (PMC7542560; doi:10.1128/mSystems.00534-20)
Supplement: TABLE S3 [file mSystems.00534-20-st003.docx]

**Table S3:**

| **Name** | **Sequence (5’ → 3’)** | **Use** |
| --- | --- | --- |
| Butters_CVR_Forward_XbaI | CCCTCTAGAAAGCATGATGCCGCGACGCCGCTATTCCGGT  TCTGCTG | To clone Butters genes *30, 31, and 30-31* into pMH94. (primer A for PCR_1 and PCR_3) |
| R_Butters_gp30_Xba1 | GGGTCTAGACTATCCACTGTCACCACCCCATCCTGCCC | To clone Butters gene *30* into pMH94 |
| F_Buttersgp31_clean | GCTACGCCACAAAGGTATAGGTGGATAGATTCAACATTG | To clone Butters gene *31* into pMH94. (primer B for PCR_2) |
| R_Buttersgp31_clean | CAATGTTGAATCTATCCACCTATACCTTTGTGGCGTAGC | To clone Butters gene *31* into pMH94 (primer C for PCR_1) |
| R_gp31_XbaI | GTCTCTAGACATTCCGTCATGCGACGAAGG | To clone Butters gene *31, and 30-31* into pMH94 (primer D for PCR_2 and PCR_3) |
| F_middle_M13_ori | TAGAGCTTGACGGGGAAAGCC | For sequencing inserts in pMH94 clones |
| P94_rev_seq_primer | TGTCGTTCACGGCTCTCAGC |  |
| F_Butters_upstream flanking_gp30 | GGCCTACTCGTCGTCAACGGCGCG | For Δgene *30* BRED recombination substrate PCR (primer #1 for PCR_1 and PCR_3) |
| R_Buttersgp30_deletion | ATCCTGCCCACAGCATACCTTTGTGGCGTAGCTCTCATG | For Δgene *30* BRED recombination substrate PCR (primer #3 for PCR_1) |
| F_Butters_gp30_deletion | ATAGATGCTGTGGGCAGGATGGGGTGGTGACAGTGGATAG | For Δgene *30* BRED recombination substrate PCR (primer #2 for PCR_2) |
| R_Butters_downstream flanking_gp31 | CTCGGCCTGGCGGTCGGCTCTTTG | For Δgene *30* BRED recombination substrate PCR (primer #4 for PCR_2 and PCR_3) |
| F_Buttersgp29 | GTGATCGCTGACGCACTGCGC | For Δgene *30* post-BRED PCR screening |
| R_Butters_gp31_stop | TCACTTTGTGGCATCAAAACCGTTGAGC |  |
| F_Butters_gp30_start | ATGCTGTGGGATCGCACATCGC | To clone Butters gene *30* into pEXP/5 |
| R_Butters_gp30_nostop | TCCACTGTCACCACCCCATCCTGCCC |  |
| F_Butters_gp31_start_RBS_XbaI | CCTCTAGAAGGAGATACCCTATGGATAGATTCAACATTGTTCCGC | To clone Butters gene 31 into pEXP5/Kan |
| R_Butters_gp31_FLAG_Stop_XbaI | GGGTCTAGATCACTTGTCGTCATCGTCTTTGTAGTCCTTTGTGGCATCAAAACCGTTG |  |
| F_sspI_Kanamycin | GGGAATATTTTGAAAAAGGAAGAGTATGAGCCATATTCAACGGGAAACGTCG | Used to isolate kanamycin gene from pENTR |
| R_Kanamycin_BanI | CCCCCCGGCGCCTTAGAAAAACTCATCGAGC |  |
| F_pJS167EcoRI | CATTCTGTGAAAGCTTAATTAGCTGATCTAGACGCGTGCTAG | To amplify ColE1 backbone |
| R_pJS167EcoRI | GGTACCTTTCTCCTCTTTAATGAATTTTCTGTGTG |  |
| F_gp21 | AGAAAATTCATTAAAGAGGAGAAAGGTACCATGGCACAAGCAGAACTGCTGGAC | To clone Butters gene 21 into ColE1/backbone |
| R_gp21T | AGCACGCGTCTAGATCAGCTAATTAAGCTTCTAACAGCAGCCCGGGCAACAG |  |
| F_gp31 | AGAAAATTCATTAAAGAGGAGAAAGGTACCATGGATAGATTCAACATTGTTCCGCTGATTC | To clone Butters gene 31 into ColE1/backbone |
| R_gp31 | AGCACGCGTCTAGATCAGCTAATTAAGCTTTCACTTTGTGGCATCAAAACCGTTGAG |  |
| R_gp31T | AGCACGCGTCTAGATCAGCTAATTAAGCTTCTAACAGCAGCCCGGGCAACACTTTG | With F_gp31, to clone Butters gene 31 with TC-tag into ColE1/backbone |
| F_gp30 | AGAAAATTCATTAAAGAGGAGAAAGGTACCATGCTGTGGGATCGCACATCGCATG | To clone Butters gene 30 with TC-tag into ColE1/backbone |
| R_gp30T | AGCACGCGTCTAGATCAGCTAATTAAGCTTCTAACAGCAGCCCGGGCAACATC |  |
| R_31T_30-31T | AATTCGCTAGTTTACTAACAGCAGCCCGGGCAACACTTTG | With F_gp31, to clone Butters gene 31 with TC-tag and gene 30 into ColE1/backbone |
| F_31T_30-30 | CGGGCTGCTGTTAGTAAACTAGCGAATTCATTAAAGAGGAGAAAGGTACCATGCTGTGGGATCGCACATCGCATG |  |
| R_31T_30-30 | AGCACGCGTCTAGATCAGCTAATTAAGCTTCTATCCACTGTCACCACCCCATCCTG |  |
| R_31_30T-31 | AATTCGCTAGTTTATCACTTTGTGGCATCAAAACCGTTGAG | With F_gp31 and R_gp30T, to clone Butters gene 31 without TC-tag and gene 30 with TC-tag into ColE1/backbone |
| F_31_30T-30 | ATGCCACAAAGTGATAAACTAGCGAATTCATTAAAGAGGAGAAAGGTACCATGCTGTGGGATCGCACATCGCATG |  |

**Supplemental References**

1. M. H. Lee, L. Pascopella, W. R. Jacobs, G. F. Hatfull. Proc. Natl. Acad. Sci. USA 88: 3111-3115, 1991.
2. R. M. Dedrick, D. Jacobs-Sera, C. Guerrero, R. Garlena, T. Mavrich, W. H. Pope, J. Cervantes Reyes, D. A. Russell, T. Adair, R. Alvey, J. A. Bonilla, J. Bricker, B. Brown, D. Byrnes, S. Cresawn, W. Davis, L. Dickson, N. Edgington, A. Findley, U. Golebiewska, J. Grose, C. Hayes, L. Hughes, K. Hutchison, S. Isern, A. Johnson, M. Kenna, K. Klyczek, C. M. Mageeney, S. Michae, S. Molloy, M. Montgomery, J. Neitzel, S. Page, M. Pizzorno, M. Poxleitner, C. Rinehart, C. Robinson, M. Rubin, J. Teyim, E. Vazquez, V. Ware, J. Washington, G. F. Hatfull. *Nature Microbiology* 2:16251, 2017.
3. S. Keppetipola, W. Kudlicki, B. D. Nguyen, X. Meng, K. J. Donovan, A. J. Shaka. J. American Chem. Soc. 12: 4508-4509, 2006.
4. S. B. Snapper, R. E. Melton, S. Mustafa, T. Kieser, W. R. Jacobs Jr. Mol Microbiol 4:1911–1919, 1990.
5. J. Stricker, S. Cookson, M. R. Bennett, W. H. Mather, L. S. Tsimring, J. Hasty. Nature 456: 516-519, 2008.
6. S. G. Cresawn, M. Bogel, N. Day, D. Jacobs-Sera, R. W. Hendrix, G. F. Hatfull

BMC Bioinformatics 12: 395, 2011.

1. A. Krogh, B. Larsson, G. von Heijne, E. L. L. Sonnhammer. J. Mol. Biol. 305:567-580, 2001.
2. E. L. L. Sonnhammer, G. von Heijne, A. Krogh, p.175-182 *in* J. Glasgow, T. Littlejohn, F. Major, R. Lathrop, D. Sankoff, C. Sensen, ed., *Proceedings of the Sixth International Conference on Intelligent Systems for Molecular Biology*, 1998.
